# Supplementary material for: Viral load non-suppression status among women exposed to Dolutegravir-based versus Efavirenz-based regimens in Ethiopia: A before-and-after study
Source: PLoS One. 2024 Jun 10;19(6):e0305331. doi: 10.1371/journal.pone.0305331 (PMC11164349; doi:10.1371/journal.pone.0305331)
Supplement: S1 Fig — (DOCX) [file pone.0305331.s001.docx]

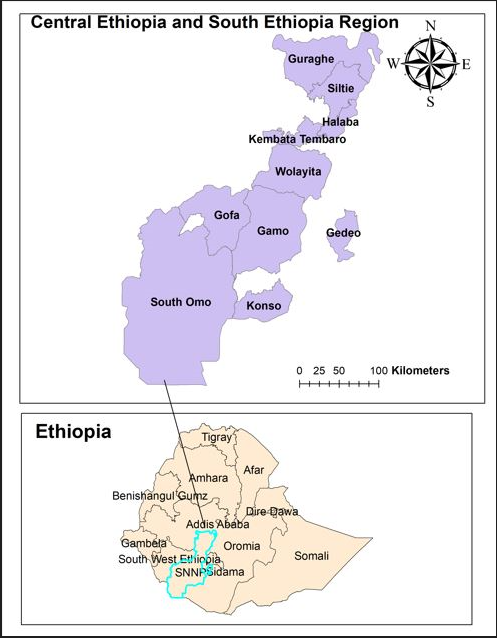


Fig. 1 Map of the study area

***Source:*** *Humanitarian Data Exchange (*[*https://data.humdata.org/dataset/cod-ab-eth*](https://data.humdata.org/dataset/cod-ab-eth)*)*

***Note:*** *This is the map of the Southern Nations, Nationalities, and Peoples’ Region (SNNPR) in which the study was conducted. Currently, SNNPR is divided into two regions (Central Ethiopia and South Ethiopia Regions) by 19 August 2023 after a successful referendum* (Wikepedia, 2023)*.*
