# Supplementary material for: Viral load non-suppression status among women exposed to Dolutegravir-based versus Efavirenz-based regimens in Ethiopia: A before-and-after study
Source: PLoS One. 2024 Jun 10;19(6):e0305331. doi: 10.1371/journal.pone.0305331 (PMC11164349; doi:10.1371/journal.pone.0305331)
Supplement: S1 File — (PDF) [file pone.0305331.s002.pdf]

ወላይታ ሶዶ ዩኒቨርሲቲ
ወላይታ ሶዶ

Wolaita Sodo University
Wolaita Sodo

WSU-Institutional Research Review Committee
የወ.ሶ.ዩ-ተቋማዊ የምርምር ስነ-ምግባር ገምጋሚ ኮሚቴ

Ref No: [illegible] /32/223

Date: 17/5/15 EC

Certificate Ref. No: WSU-IRRC/002/2023

Subject: **Ethical clearance certificate**

The research project entitled "**Viral load non-suppression status among women exposed to Dolutegravir-based versus Efavirenz-based first-line antiretroviral therapy in southern Ethiopia: A retrospective before-after study**" has been reviewed by IRB of Wolaita Sodo University and found to be ethically accepted. Thus, the committee has authorized the principal investigator Mr Wolde Facha to commence his project accordingly.

Elements Approved:

1. Protocol/proposal :
2. Informed Consent form (if applicable):
3. Information sheet (if applicable) :
4. Assent form (if study include under 18 year or unconscious group): N/A
5. Community consent (if applicable): N/A

The investigator is expected to critically follow and fulfill the following obligates and responsibilities. The Investigator:

1. Should comply with the standard of national and international scientific and ethics guidelines
2. All amendments and changes made in protocol and consent form after the ethical approval is secured must be approved by the IRB of the University
3. The principal investigator should submit follow-up report every three month until completion
4. The final work should be submitted the IRB of the University

N.B. This ethical clearance is not valid for material (specimen) transfer unless approved by the NERC

Institutional Review Board (IRB) approval period: January 25, 2023 to Feb 25, 2024

Chairman

Name: Dr. Takele Teklu

Signature: [signature]

Secretary

Name: [illegible] Alembo (PHD)

Signature: [signature]

Director ,Research Affairs Directorate

መልሱን ሲጽፉልን እባክዎን የእኛን ቁጥር ይጥቀሱ

✉ 138
E-mail:-wsuniv@ethionet.et

☎ 251-046-551-19 11
Fax: +251-046-551-5113

CamScanner
