# Supplementary material for: Viral load non-suppression status among women exposed to Dolutegravir-based versus Efavirenz-based regimens in Ethiopia: A before-and-after study
Source: PLoS One. 2024 Jun 10;19(6):e0305331. doi: 10.1371/journal.pone.0305331 (PMC11164349; doi:10.1371/journal.pone.0305331)
Supplement: Attachment — Submitted filename: Point-by-point response to reviewer on VLS.docx [file pone.0305331.s005.docx]

1. **A point-by-point response to the Editor’s Comments for Manuscript ID PONE-D-24-09455**

**Dear Dr Melissa Hardgrave,**

Thank you for your constructive feedback on our manuscript "***Viral load non-suppression status among women exposed to Dolutegravir-based versus Efavirenz-based first-line antiretroviral therapy in Southern Ethiopia: A retrospective before-after study***" submitted to **PLOSE ONE**. The authors are happy to get the opportunity to revise the manuscript to increase its quality. The authors have carefully considered each of the comments provided and have addressed them as follows:

| **Comment N^o^** | **Editor’s comment** | **Authors’ response** |
| --- | --- | --- |
| 1 | Please ensure that your manuscript meets PLOS ONE's style requirements, including those for file naming. | Dear Editor, thank you for your constructive suggestions to align the manuscript with PLOSE ONE’s style. The authors revised the content of the manuscript to meet PLOSE ONE’s style. |
| 2 | Your ethics statement should only appear in the Methods section of your manuscript. If your ethics statement is written in any section besides the Methods, please move it to the Methods section and delete it from any other section. Please ensure that your ethics statement is included in your manuscript, as the ethics statement entered into the online submission form will not be published alongside your manuscript. | Dear Editor, thank you for your comments. The authors moved ethics statements to the methods section and deleted them from the other section as per the editor’s suggestion. |
| 3 | We note that Figure 1 in your submission contains map images that may be copyrighted. All PLOS content is published under the Creative Commons Attribution License (CC BY 4.0), which means that the manuscript, images, and Supporting Information files will be freely available online, and any third party is permitted to access, download, copy, distribute, and use these materials in any way, even commercially, with proper attribution. For these reasons, we cannot publish previously copyrighted maps or satellite images created using proprietary data, such as Google software (Google Maps, Street View, and Earth). We require you to either (1) present written permission from the copyright holder to publish these figures specifically under the CC BY 4.0 license, or (2) remove the figures from your submission: | Dear Editor, the authors appreciate your concern about copyright issue for the study area map. The shape file was accessed from the Humanitarian Data Exchange website (<https://data.humdata.org/dataset/cod-ab-eth>). Since the shape file is in the public domain and can be used freely, there is no need to have written permission from anyone else to publish these figures under the CC BY 4.0 license. |
| 4 | Please include captions for your Supporting Information files at the end of your manuscript, and update any in-text citations to match accordingly. | Dear Editor, thank you for your reminder. The authors included captions for the Supporting Information files at the end of the manuscript and updated in-text citations to match accordingly. |

Thank you for your continued support.

Warm regards,

Wolde Facha (Corresponding author)

2. **A point-by-point response to Reviewer 1**

Dear reviewer, the authors appreciate your positive view regarding the manuscript. For the comments and suggestions you have given, please see the authors’ responses below. Thank you very much.

| **Comment N^o^** | **Reviewer’s comment** | **Authors’ response** |
| --- | --- | --- |
| 1 | **Abstract:**  Well summarised with a clear description of the subtitles. Is it worth mentioning WHO recommendations in the second sentence in the background here? | Dear Reviewer, your concern is right. The authors rewrote the second sentence of the background in the abstract section, including the phrase ‘WHO recommendation’ as per the suggestions. |
| 2 | **Introduction:**  I think this was well written. | Dear Reviewer, thank you for your positive feedback on the introduction section. |
| 3 | **Materials and Methods:**  The title of the study says the region sampled was in Southern Ethiopia but under study settings, two regions are mentioned, both Central and Southern Ethiopia. | Dear Reviewer, thank you for your comment. Central Ethiopia and South Ethiopia were two regions found in southern part of Ethiopia. However, to avoid such confusion by readers, especially outside Ethiopia, the authors edited the name of the study setting in the revised manuscript. |
| 4 | **Results:**  The analytical approach of the results is clear. | Dear Reviewer, the authors acknowledge your witness for the clear analytical approach the authors conducted. |
| 5 | **References:**  Satisfactory | Dear Reviewer, thank you for your positive feedback for the reference section. To be most appropriate, the authors edited the reference section as per PLOSE ONE’s guidelines. |

3. **A point-by-point response to Reviewer 2**

| **Comment N^o^** | **Reviewer’s comment** | **Authors’ response** |
| --- | --- | --- |
| 1 | General comment |  |
| 1.1. | The manuscript is generally reasonably written, but there are some major methodological issues, including in the study design, sampling, and data analysis that need review and address. | Dear Reviewer, thank you for your positive outlook regarding the manuscript. Hence, as per the reviewer’s comments, all the methodological issues raised were sufficiently addressed in the revised manuscript. |
| 1.2 | My overall recommendations are that the authors need to do a major review of the manuscript, especially the study design, sampling and data analysis of the methods, and presentation of the results. The manuscript also needs some editing in addition to rewriting some sections to improve grammar and language for better flow. | Dear Reviewer, thank you for your constructive comments. The authors rewrote the method section of the manuscript as suggested by the reviewer. Besides, the manuscript was edited, and respective sections were rewritten to improve grammar and language for better flow. |
| 2. | Examples and evidence |  |
| 2.1. | Major issues |  |
| 2.1.1 | ***Overall study context:*** The authors provide the status of HIV and PMTCT, which seems to be the global situation, but they do specifically indicate this. They should mention that the data is global, and they should also provide the situation in Ethiopia. Furthermore, the data provided for children 0-14 years who acquired HIV seems to be for the year 2022, and the statement will need correction. | Dear Reviewer, your idea is correct. The data presented in the first paragraph of the introduction section is global data. It is mentioned in the revised manuscript as per the comment. Concerning the year, the 2022 data is released by August 2023, and the 2023 data will be released by August 2024 (i.e. after three months). Therefore, the most recent and available data for citation is the 2022 data. |
| 2.1.2 | ***History of studies on effect of regimens on VLS:*** The following statement in the abstract and introduction, “However, its effect on VLS has not been investigated” seems to be a broad claim. It would be more accurate to say that it has not been investigated in the specific context of the study, which is among women in Southern Ethiopia or in the PMTCT care. | Thank you for your constructive comment to avoid a broad claim. The authors revised the sentence and edited it in the revised manuscript claiming that the VLS has not been investigated among women in PMTCT care in Ethiopia. |
| 2.1.3 | ***Effectiveness of DTG-based regimens:*** The sentence in the introduction, starting “However, the effectiveness of DTG-based regimens ….” may not be correct. Five African countries (Eswatini, Botswana, Rwanda, Tanzania and Zimbabwe) have already reached 95-95-95. (Reference: “The path that ends AIDS: UNAIDS Global AIDS Update 2023”. Geneva: UNAIDS; 2023). | Dear Reviewer, thank you for sharing evidence on the achievement of the third 95 targets by five sub-Saharan African countries. The authors explained this concept in the revised manuscript. |
| 2.1.4 | ***Issue with the study design:*** The study design is not clearly defined. A retrospective before-after study design typically involves comparing outcomes before and after an intervention in the same group. However, in this case, it seems like two different groups (those on EFV-based ART and those on DTG-based ART) are being compared. These seem to be different sets of patients. This means that the same patient cannot be in both groups. This is therefore more akin to a cohort study. Please clarify the study design and consider revising it if necessary. | Dear Reviewer, the authors would appreciate your critical view on the study design that the authors used (before-and-after). As the reviewer indicated, two different groups (those on EFV-based ART and those on DTG-based ART) were compared by reviewing records of eight-year data retrospectively. However, unlike the retrospective cohort study, the two groups were not followed concurrently in our study. This was because of the program shift from EFV-based regimens (the before group) to DTG-based regimens (the after group). Thus, the authors used an uncontrolled before-and-after study. It is a non-randomised design conducted to evaluate the effects of an intervention by comparing the outcomes of study participants investigated before the intervention with those measured afterward (Sedgwick, 2011, 2014). Besides, the design is preferred to evaluate the effectiveness of a program that was implemented one after the other on different participants (Sedgwick, 2011). In our case, VL non-suppression status was compared among women on EFV-based regimens (the before group) with the DTG-based regimens (the after group) to evaluate the effectiveness of the new drug regimens (the DTG-based regimens) over the previously used EVF-based regimens. As the before-and-after design offers better evidence of intervention effectiveness than other non-experimental designs (Sedgwick, 2011), the authors preferred the uncontrolled before-and-after study design to the retrospective cohort design. |
| 2.1.5 | ***Use of health facility as randomization unit:*** The authors mention that “…. facilities ...…. were randomly selected to be included in the study”. The method or process of randomization is not clear. Was it simple randomization, block randomization, or stratified randomization? As it seems the randomization unit is the clinic, and since the method or process of randomization was not described, in the event that randomization was not effective, issues that could arise and need address could include: (a) selection bias if the population attending each clinic differs significantly in characteristics related to the outcome of interest, e.g age difference in between clinics, difference in health outcomes due to underlying population differences between the clinics; (b) confounding if there are any unmeasured confounding variables that differ between clinics and affect the outcome, e.g. quality of care, experience level of the staff of availability of certain resources; (c) cluster effects if patients within the same clinic are more similar to each other than to patients in other clinics; (d) problems with generalizability to all clinics or populations if the clinics included had specific characteristics that make them not representative of all clinics. There is therefore need to clearly describe the randomization method and process to understand if the above issues may have arisen. | Dear Reviewer, the authors appreciate your concern about how randomization was performed. Currently, a total of 140 facilities (49 hospitals and 91 health centers) are providing PMTCT and ART services in separate units. However, 72 facilities (40 hospitals and 32 health centers) have been providing both PMTCT and ART services since 2015, when the study period started. Besides, the seats of zones and special districts in the study area are categorized into nine high-, ten medium-, and three low-priority towns based on the incidence of HIV (FMoH, 2023).  Facilities were stratified by facility type (hospitals and health centers) and priority towns (high, medium, and low) based on the incidence of HIV (FMoH, 2023). Since facilities under each category are similar in terms of service quality, women’s characteristics, and level of staff providing the service, the authors employed stratified random sampling methods to select facilities. Thus, nearly half of the facilities (20 hospitals and 14 health centers) from the nine high-priorities and ten medium-priority towns were included in the study. All women who fulfilled the eligibility criteria from selected facilities were included in the study to get an adequate sample. This is explained in the revised manuscript. However, if you mean randomization to intervention/control arms, there was no such randomization as the study did not involve a randomized trial. |
| 2.1.6 | ***Use of consecutive sampling:*** The authors mention that they used a consecutive sampling technique, but it’s not clear how this was implemented. Did they include all eligible participants until they reached their sample size? It is important to note that consecutive sample can introduce sampling bias depending on how it is done. It is therefore important for the authors to provide more details on how it was done and how they ensured that bias was not introduced. Furthermore, as the source of participants is an electronic database, the authors could consider randomly selecting participants from the data base instead of using consecutive sampling, which would ensure full randomization. | Dear Reviewer, thank you for raising clarity question regarding the sampling technique. In this study, the total number of pregnant and breastfeeding women receiving PMTCT care was 1,236 (675 in South Ethiopia and 561 in Central Ethiopia), and 1,063 (86% of women) are getting PMTCT service at the selected 34 facilities. Thus, the authors included all eligible participants starting from those discharged from PMTCT care in the most recent period and went backward in time selecting study participants serially until the required sample size was met. |
| 2.1.7 | ***Threshold for covariate selection into the model:*** The authors selected covariates for entry into the multivariable model based on a p-value of less than 0.25 in the unadjusted analyses, which is not a standard approach as it may lead to over fitting. Though a more stringent or more conservative thresholds than the standard 0.05 can be used depending on the context, other factors may need to be taken into account as depending only on a p value can result in over fitting or under fitting of the model. Other statistical considerations that could be taken into account include the effect size, confidence intervals, and the clinical or practical significance of the covariates. It would therefore be helpful to provide a rationale for the choice of the threshold and what other consideration were taken into account. | The authors appreciate this critical comment. The authors considered a p-value as of less than 0.25 in the unadjusted analysis to enter variables into multivariate analysis(Bursac *et al.*, 2008). When a variable with a p-value of 0.05 was entered, the log-likelihood result was -401.06672 and when variables with a p-value of 0.25 were entered the log-likelihood ratio result was -398.16358. A model with a lower absolute log-likelihood value is a better fitting model than one with higher absolute log-likelihood values. Thus, the authors considered covariates with a p-value of less than 0.25 in the unadjusted analysis to get a better fitting model. Besides, maternal educational status is considered for practical importance and entered into the multivariate model (Bursac *et al.*, 2008). However, it was not significantly associated with the outcome variable. |
| 2.1.8 | ***Adherence categorization:*** The categorization of adherence into poor, fair, and good is not clearly defined. It would be helpful to provide specific criteria or thresholds for these categories. | Dear Reviewer, thank you for your constructive comment on adherence categorization. Mothers’ ART adherence was categorized as *poor*, *fair*, or *good*. Women with poor adherence status at any time during the follow-up period were classified as having *poor adherence*. It is considered poor if a woman missed greater than or equals to 5 out of the 30 doses or greater than or equals to 10 out of the 60 doses at any time during the follow-up period. A woman whose adherence status was recorded as fair (but not poor at any time during the follow-up period) was classified as having *fair adherence*. This means that a woman missed 2–4 of the 30 doses or 4–9 of the 60 doses. On the other hand, a woman whose adherence status was recorded as good (but not poor or fair at any time during the follow-up period) was classified as having *good adherence*. It was recorded as good if a woman missed only one out of the 30 doses or less than or equals to three out of 60 the doses (FMoH, 2021a, 2021b). The authors referred readers to get details of adherence categorization in the revised manuscript. |
| 2.1.9 | ***Missing data:*** The results do not mention any missing data. Considering this study is using routine data, it would be very unusual for there to be no missing data. It’s therefore crucial to address this in the results and discuss how it might have affected the findings. However, if there actually were no missing data, the authors would need to explain how that was achieved. | Dear Reviewer, the authors would like to appreciate your comment to explain how we dealt with missing data. The authors approached a total of 1,063 women on PMTCT care, and 139 (13.1%) participants’ records were excluded from the final analysis due to missing data. Thus, the study included 924 women (462 exposed and 462 unexposed) enrolled in PMTCT care. This is explained in the result section of the revised manuscript. The issue of missing data effect on the result is explained in the limitation section. |
| 2.1.10 | ***Exclusion of variables statistically significant in table 4:*** Only the seven statistically significant variables are included in table 4. The authors could consider including those that were not statistically significant in table 4. | Dear Reviewer, thank you for your comment. The authors included all variables entered into multivariate analysis in Table 4 as per the suggestion. |
| 2.1.11 | ***Confounding factors:*** The results do not discuss potential confounding factors. For example, were there any differences in the health status, lifestyle, or other characteristics between the two groups (DTG-based regimens arm and EFV-based regimens arm) that could have influenced the results? | The authors recognize the reviewer’s concern. However, the two groups have almost similar study participants regarding number, health status, lifestyle, and other characteristics, as shown in Tables 1-3. Besides, the two groups (DTG-based regimens arm and EFV-based regimens arm) got PMTCT services from the same facility, with similar providers by profession. Besides, the authors conducted a stratified analysis to check the existence of potential confounding variables like residence and facility types. However, there were no confounding variables included in the study since the crude result does not differ from the adjusted result. In this study, training (the variable that was not included in the study) might be a potential confounder since service delivery by trained and non-trained providers might affect the outcome. As the authors used retrospective data, it was difficult to assess whether the service was delivered all the time by trained providers or not. This is mentioned in the limitation section of the revised manuscript  . |
| 2.1.12 | ***Inconsistent comparisons:*** The authors mentioned that the study findings are in line with the China study (second discussion paragraph), and indicate the comparison regimen was protease-based, and put in brackets EFV-based. The China study was actually about low-level viremia and virological failure, and the comparison group was protease-based. The China study may therefore not be appropriate as a comparison group. Furthermore, viral load suppression was also compared with those of studies conducted in different settings using different viral load suppression thresholds, but they do not seem to take into account potential differences in study populations, methodologies or healthcare systems. | Dear Reviewer, thank you for your critical observation. Integrase inhibitors (DTG-based regimens) demonstrated a protective effect against low-level viremia events in a Chinese study. However, the comparator group included Protease inhibitors but not non-nucleoside reverse transcriptase inhibitors (EFV-based regimens). Therefore, as the reviewer indicated, the study conducted in China is not appropriate as a comparison group, and thus the authors excluded this study from discussion in the revised manuscript. |
| 2.13 | ***Unexplained contradictions:*** The authors state that older women were more likely to have non-suppressed viral load status but cite studies that found the opposite. The manuscript does not explain or discuss why there were these contradictions. | Dear Reviewer, thank you for your understanding. This study claimed that ‘Older women were approximately 1.38 times at higher risk of having non-suppressed VL status than younger women’. This means that older women are more likely to have treatment failure than younger ones, which is in line with the references mentioned in the discussion section. The reference the authors used on paragraph four in the discussion section reported that ‘Immunological failure was 1.78 times more likely among the older age group (≥40 years) than younger ones (<40 years). |
| 2.2 | . Minor issues |  |
| 2.2.1. | ***The following statements in the introduction may need review:*** |  |
|  | A) “Failure to achieve adequate VLS status ….. the major risk factor ….” does not flow well and may need to be rewritten. It is usually high viral load that is the risk factor for MTCT of HIV. | Dear Reviewer, thank you for your comment. The sentence ‘Failure to achieve adequate VLS status … the major risk factor …” is rewritten for better understanding as per the reviewer’s suggestion in the revised manuscript. |
|  | B) In the second paragraph and first line of the introduction: It may be better to add to this phrase “putting women on ART, preferably DTG-based regimens” to make the sentence complete. | Thank you for your input. The authors added the phrase ‘putting women on ART, preferably DTG-based regimens’ as per the reviewer’s suggestions to complete the sentence. |
| 2.2.2. | ***Rationale for choosing study sites:*** The study setting is described, but the rationale for choosing these specific regions and zones is not clear. It would be helpful to provide a brief explanation for this choice. | The study area is administratively divided into 19 zones (the largest administrative structure in the region) and three special districts (Wikepedia, 2023b, 2023a). The seats of these zones and special districts are labelled into nine high-, ten medium- and three low-priority towns based on the incidence of HIV. Those towns with HIV incidence of ≥0.03%, 0.01- 0.029%, and < 0.01% were categorized as high-, medium-, and low-priority (FMoH, 2023). All the low-priority areas either have not started the PMTCT service since September 2015 (when the study started) or have a very low number of women (below 18 women) in PMTCT care, thus were excluded due to the unavailability of data for the whole study period or resource limitations. Thus, the authors included ten medium-priority towns and nine high-priority towns to get the desired number of study participants. Thirty-four facilities (20 hospitals and 14 health centers) from the nine high-priority and ten medium-priority towns were selected among 72 facilities that have been offering PMTCT services since 2015. This is described in the revised manuscript. |
| 2.2.3. | ***Assumptions for sample size calculation:*** The sample size calculation is generally clear, but it would be helpful to provide more details about the assumptions used. For example, how was the ratio of unexposed to exposed determined to be one? Also, it would be helpful to provide a reference or explanation for the decision to add 20% for missing data. | The ratio of one-to-one is based on different literatures (Kouanfack *et al.*, 2019; Kintu *et al.*, 2020; Mahale, Patel and Kasmani, 2023) and the existing eligible study participants in two groups after the implementation of DTG-based regimens. Their numbers are almost equal in the two groups. The use of 20% for missing data is based on Regional Health Bureau PMTCT experts’ suggestions to compensate for the expected missing data for the variables included in the study. This is explained in the revised manuscript. |
| 2.2.4. | ***Operational definitions:*** The operational definitions are clear, but it would be helpful to provide a bit more context. For example, why is a viral load count of less than or equal to 50 copies/ml considered suppression as it is not the standard definition as recommended by WHO (less than or equal 1,000 copies/ml). If the authors still prefer to use the non-standard cut-off for the study, it would be helpful to provide the specific rationale. | Dear Reviewer, thank you for your clarity questions. A viral load that is undetectable, or less than or equal to 50 copies/ml considered as viral suppression according to the revised national guidelines of Ethiopia.  The guidelines also categorize viral load status as low-level viremia if one or more viral load results are detectable (more than 50 copies/ml) but equal to or less than 1000 copies/ml, and as a virological failure if viral load is above 1000 copies/ml based on two consecutive viral load measurements in three months apart with enhanced adherence support following the first viral load test (FMoH, 2021a, 2022b). The cut-off point is explained in the operational definition section of the revised manuscript. Besides recent studies use 50 copies/ml as the cut-off point for suppressed viral load (Duiculescu *et al.*, 2013; Kouanfack *et al.*, 2019; Malaba *et al.*, 2022) |
| 2.2.5 | ***Referencing:*** This is issues with some of the referencing. For example, reference [10] is cited twice but it’s not clear if it refers to the same source. | Dear Reviewer, thank you for your comments on the reference section. Reference [10], the PMTCT training manual of the 2021 edition, is not cited twice. Maybe editions 2017 and 2021 are mentioned in the reference section separately to show the DTG-based regimens change from the EFV-based regimens. |
| 2.2.6. | ***Study setting:*** The authors mention that details on study setting are provided elsewhere. It would be beneficial to include at least a brief summary in this paper for completeness. | Dear Reviewer, thank you for your concern about the study setting. A brief summary of the study setting is included in this study and is explained in the revised manuscript. |
| 2.2.7. | ***Effect of methodological limitations on results:*** The paper acknowledges methodological limitations. It however does not discuss how these limitations might have affected the results. | Dear Reviewer, thank you for your concern. The methodological limitations i.e. measurement and recording errors as well as exclusion of missing data might result in underestimation or overestimation of viral load non-suppression. This is reported in the revised manuscript. |
| 2.2.8. | ***Lack of clarity on the study outcome:*** Though the title of the study clearly indicates it is on viral load non-suppression, some parts of the manuscript refer to viral load suppression so that the message of the study outcome is not consistent. It will help to harmonize this throughout the manuscript. | Dear Reviewer, the authors agree with your suggestion to be consistent with the study outcome throughout the manuscript. However, in some sentences, it is difficult to omit the words ‘viral load suppression’ totally from the manuscript. For instance, the WHO recommendation to use DTG-based regimens is to achieve viral load suppression of 95% and above among women on PMTCT care. Thus, in some parts of the manuscript, the effect of DTG-based regimens is better explained in terms of achieving ‘viral load suppression’ than the ‘viral load non-suppression’ to be in line with the national and global targets. Other sentences that need to be mentioned as viral load non-suppression have been edited as per the reviewer’s suggestion throughout the manuscript. |
| 2.2.9. | ***Numbers in the results section:*** Most of the numbers and percentages in the tables are repeated in the text. It would help readability and flow if only essential numbers and percentages are mentioned in the text with references being given to the tables. The results sections other than the one on factors associated with VL non-suppression do not refer to the variables found to meet the 0.25 p-value threshold for inclusion in the multivariate analysis. | Dear Reviewer, thank you for your constructive comments not to repeat the numbers and percentages in the text with references being given to the tables. The authors accepted the reviewer’s recommendation and avoided repetition in the revised manuscript. The authors also appreciate the reviewer’s concern about not referring the variables that found to meet the 0.25 p-value threshold in multivariate analysis. If the reviewer’s suggestion is based on Table 1-3 Pearson’s chi-squared value, it is used to describe the proportion of women in the EFV-based versus DTG-based regimens but not to select candidate variables for multivariate analysis. Candidate variables for multivariate analysis were selected based on the p-value result between the covariates and the outcome variable (suppressed versus non-suppressed viral load status) as reported in Table 4 and based on practical importance for some variables. |
| 2.2.10. | ***Categorization of daily labourers as high risk:*** It is not clear why daily labourers are included in the high-risk group. The authors may need to explain this in the Ethiopian context. | Thank you for your operational questions on daily laborers in the Ethiopian context. These are a group of population who do not have permanent working environments at any of the governmental, non-governmental, or private organizations. They are mobile workers moving from one place to another place seeking work to get money for daily consumption. There is a high incidence of HIV infection among this group of people and, as a result, they are categorized as a high-risk population group like commercial sex workers and long-distance truck drivers in the Ethiopian context (FMoH, 2022a). |
| 2.2.11. | ***Language and Grammar:*** There are some typos in the document and statement that need to be revised, e.g. “DGT-based regimen” and “Camerron” in the sample size section. There are also words that may need to be replace, for example: “adequate” in the abstract background, “employed” in the abstract methods, “supplementation” in the abstract conclusion; “besides” in the results section on factors associated with VL non-suppression; and the word “direction” in the third paragraph of the introduction section could be replaced with “Global targets”. There is also need for consistency in the use of some abbreviations, e.g. both “VLS” and “VL status” which could be harmonized throughout the manuscript. Overall, many of the sections do not flow well and need language and technical editing to flow better. | Dear Reviewer, thank you for your comments that need typo revisions in the document. The authors accepted the reviewer’s comments and revised all indicated words and phrases in the revised manuscript. Regarding consistency in the use of some abbreviations like “VLS” and “VL status”, the authors harmonized the abbreviations throughout the revised manuscript. Dear reviewer, your concern about language and technical editing for better flow of the sentences is also appropriate. The revised manuscript has now been thoroughly edited for language and for flow. |
| 2.2.12. | ***Strengthening the conclusion:*** The conclusion could be strengthened by summarizing the main findings of the paper and discussing their implications for clinical practice and future research. It would also be helpful to highlight key study limitations and their impact on the results. | Dear Reviewer, thank you for the suggestions on the conclusion part. The authors revised the conclusion part by summarizing the main findings and their implications for clinical practice and future research in the revised manuscript. The authors also highlighted key study limitations and their impact on the results in the revised manuscript. |

# References

Bursac, Z. *et al.* (2008) ‘Purposeful selection of variables in logistic regression’, *Source Code for Biology and Medicine*, 3, pp. 1–8. doi: 10.1186/1751-0473-3-17.

Duiculescu, D. *et al.* (2013) ‘Dolutegravir plus Abacavir–Lamivudine for the Treatment of HIV-1 Infection’, *New England Journal of Medicine*, 369(19), pp. 1807–1818. doi: 10.1056/nejmoa1215541.

FMoH (2021a) ‘National comprehensive HIV prevention, care, and treatment training participant manual’, *FMoH*, p. 158.

FMoH (2021b) ‘National comprehensive PMTCT/MNCH integrated training manual’. Addis Ababa: FMoH, pp. 368–370.

FMoH (2022a) ‘National Comprehensive HIV Testing Service Training Manual’. Addis Ababa, p. 216.

FMoH (2022b) ‘National guidelines for comprehensive HIV prevention, care and treatment’. Addis Ababa: FMoH, p. 262.

FMoH (2023) ‘HIV National Strategic Plan 2023/24- 2026/27’. Addis Ababa, p. 156.

Kintu, K. *et al.* (2020) ‘Dolutegravir versus efavirenz in women starting HIV therapy in late pregnancy (DolPHIN-2): an open-label, randomised controlled trial’, *The Lancet HIV*, 7(5), pp. e332–e339. doi: 10.1016/S2352-3018(20)30050-3.

Kouanfack, C. *et al.* (2019) ‘Dolutegravir-Based or Low-Dose Efavirenz–Based Regimen for the Treatment of HIV-1’, *New England Journal of Medicine*, 381(9), pp. 816–826. doi: 10.1056/nejmoa1904340.

Mahale, P. R., Patel, B. S. and Kasmani, N. (2023) ‘Treatment Outcomes of Dolutegravir- Versus Efavirenz-Based Highly Active Antiretroviral Therapy Regimens Among Treatment-Naive People Living With HIV’, *Cureus*, 15(6). doi: 10.7759/cureus.40139.

Malaba, T. R. *et al.* (2022) ‘72 weeks post-partum follow-up of dolutegravir versus efavirenz initiated in late pregnancy (DolPHIN-2): an open-label, randomised controlled study’, *The Lancet HIV*, 9(8), pp. e534–e543. doi: 10.1016/S2352-3018(22)00173-4.

Sedgwick, P. (2011) ‘Non-randomised studies’, *BMJ*, 5741(343), pp. 1–2. doi: 10.1136/bmj.d5741.

Sedgwick, P. (2014) ‘Before and after study designs’, *BMJ*, 5074(August), pp. 1–2. doi: 10.1136/bmj.g5074.

Wikepedia (2023a) *Central Ethiopia Regional State - Wikipedia*. Available at: https://en.wikipedia.org/wiki/Central_Ethiopia_Regional_State.

Wikepedia (2023b) *South Ethiopia Regional State*. Available at: https://en.wikipedia.org/wiki/South_Ethiopia_Regional_State#cite_note-3.
